# Views of patients and professionals about electronic multicompartment medication devices: a qualitative study

**DOI:** 10.1136/bmjopen-2016-012915

**Published:** 2016-10-17

**Authors:** Jill Hall, Christine Bond, Moira Kinnear, Brian McKinstry

**Affiliations:** 1 Usher Institute of Population Health Sciences and Informatics, University of Edinburgh, Edinburgh, UK; 2 Institute of Applied Health Sciences, University of Aberdeen, Aberdeen, UK; 3 Pharmacy Service, NHS Lothian, Edinburgh, UK

**Keywords:** medication device, patient adherence, reminder systems, QUALITATIVE RESEARCH

## Abstract

**Objectives:**

To explore the perceived acceptability, advantages and disadvantages of electronic multicompartment medication devices.

**Design:**

Qualitative study using 8 focus groups and 10 individual semistructured interviews. Recordings were transcribed and analysed thematically. Strategies were employed to ensure the findings were credible and trustworthy.

**Participants and setting:**

Community pharmacists (n=11), general practitioners (n=9), community nurses (n=12) and social care managers (n=8) were recruited from the National Health Service (NHS) and local authority services. Patients (n=15) who were current conventional or electronic multicompartment medication device users or had medication adherence problems were recruited from community pharmacies. 3 informal carers participated.

**Results:**

Electronic multicompartment medication devices which prompt the patient to take medication may be beneficial for selected individuals, particularly those with cognitive impairment, but who are not seriously impaired, provided they have a good level of dexterity. They may also assist individuals where it is important that medication is taken at fixed time intervals. These are likely to be people who are being supported to live alone. No single device suited everybody; smaller/lighter devices were preferred but their usefulness was limited by the small number/size of storage compartments. Removing medications was often challenging. Transportability was an important factor for patients and carers. A carer's alert if medication is not taken was problematic with multiple barriers to implementation and no consensus as to who should receive the alert. There was a lack of enthusiasm among professionals, particularly among pharmacists, due to concerns about responsibility and funding for devices as well as ensuring devices met regulatory standards for storage and labelling.

**Conclusions:**

This study provides indicators of which patients might benefit from an electronic multicompartment medication device as well as the kinds of features to consider when matching a patient with a device. It also highlights other considerations for successful implementation including issues of responsibility, regulation and funding.

**Strengths and limitations of this study**

- The strength of the study lies in the inclusion of a wide range of stakeholders who had experience of either conventional or electronic multicompartment medication devices (eMMDs).
- This experience, combined with the opportunity to handle and use the devices ensured the data were based on actual experiences and contemporaneous feedback.
- Limitations of the study were the under-representation of carers, the majority of participants being professionals and that all patients were already using a MMD or eMMD.

## Background

Medication adherence has been identified as a global issue; non-adherence can have serious consequences including hospitalisation and death.^1–7^ Non-adherence is multifactorial but it is sometimes classified as unintentional, for example, practical problems with the regimen, poor instructions or poor memory, and at other times intentional due to a patient's disagreement with the need for treatment or to avoid perceived side effects.^2^ ^8^ ^9^ It is more likely in people who take several different medicines such as those living with multiple morbidities requiring polypharmacy.^10^

For people who forget to take their medicines, or get confused about them, a variety of interventions are available to support them, including multicompartment medication devices (MMDs). These range from simple pillboxes and calendar blister packs to more complex electronic devices. Trials exploring the effectiveness of preloaded calendar blister packs have yielded heterogeneous results.^11–14^ A Cochrane review of reminder packaging concluded that this may improve adherence for patients with selected conditions but further research is needed.^15^ However, the review included reminder packaging such as blister packs and reminder charts as well as MMDs and not all of the patient groups were analogous to older people taking multiple medicines. Recent research and professional guidance has also highlighted the importance of multidisciplinary assessment prior to the provision of an MMD, as well as patient involvement and availability of appropriate advice and support.^16–18^

The choice and functionality of electronic MMDs (eMMDs) is increasing rapidly. Some can prompt when to take a medication using audible and/or visual signals, dispense medications at appropriate times, give instructions to the patient, and contact a caregiver (usually by mobile internet or text to a carer) if medications are not removed. The possible benefits, such as more effective medicines use, improved patient quality of life and reduced waste, are promoted by manufacturers and mentioned in government policy.^19^ However, a recent review of the literature suggests that while eMMDs have the potential to improve adherence, the context, usability and medical condition influence their usefulness and further research is required.^20^ ^21^ Little is known about patients' and professionals' views of, and experiences with these devices. The aim of this study (which is part of a broader programme of work) was to explore the perceived acceptability, advantages and disadvantages of eMMDs.

## Methods

### Design

A qualitative design was adopted including focus groups with patients, carers and professional stakeholders, supplemented by individual interviews according to participant preference. The study was undertaken in one National Health Service (NHS) Health Board in Scotland, UK.

### Ethics and governance

The study received approval from NHS research ethics and research management approval from the relevant NHS Board. Written consent was obtained from all participants.

### Participant selection

#### Professionals

Invitation packs comprising an introductory letter, information sheet and consent form were mailed to a purposive sample of 25 community pharmacists in the NHS Board to recruit up to 10 pharmacists from a range of settings and provision of MMDs. Sampling criteria were: type of pharmacy (independent contractor/local chain/national multiple), location (urban/rural), deprivation (based on the post code of the pharmacy)^22^ and individual characteristics of the pharmacist (age/gender). General practitioners (GPs), community nurses and social care managers were initially approached through the local research network and council telecare leads, respectively, but failed to recruit any participants. Personal contacts of the research team were then followed up and a snowballing technique adopted to reach the target sample size of 20 GPs/community nurses and 5 social care managers, considered likely to achieve data saturation.

#### Patients and carers

Patient inclusion criteria were: aged 16 years or over; current user of an MMD or eMMD; or people with adherence problems identified from pharmacy medication records. Patients who were unable to provide informed consent or had a score of 24 or below on the Mini Mental State Examination were excluded.^23^ Fifteen of the aforementioned 25 community pharmacies from one NHS Board agreed to mail or hand out study invitation packs to potentially eligible patients. Those participants who expressed an interest were visited by the researcher to seek consent to participate and confirm eligibility. A maximum variation sample of 15 participants (and their lay carers if relevant) was sought based on age, gender and deprivation status of the pharmacy. For eligible participants, the researcher administered the Morisky 8-item Medication Adherence Questionnaire, the Rolyan 9-hole Peg Board Test to describe participant dexterity and the Bailey Lovie Reading Test to describe visual acuity characteristics.^24–26^ Participants were invited to attend either a focus group or interview.

### Data collection

Focus groups (or individual, semistructured interview for those unable to attend) were held between September 2014 and March 2015. A topic guide (see online supplementary appendix 1) was developed based on the research questions and refined iteratively as data collection progressed. Usability testing of a range of devices was conducted with all participants using a ‘think aloud’ technique. Devices included were those that could store multiple doses of medication and alert the patient when medication was due to be taken using audio or visual alerts and/or had the facility to alert a carer if medication had not been taken. Devices were identified from a systematic review of the literature and by other relevant local experts^20^ (see table 1). Opinions were primarily sought on the presented devices but participants also had the opportunity to discuss their own devices. Focus groups with professionals were mainly held in university locations or their work place and those with patients and carers were held at a location external to the university. Professional interviews were held in their work place and patients in their own home. All focus groups and interviews were audio-recorded and field notes were taken. They were conducted by JH, a female researcher with an MSc (including training in qualitative research methods), experience conducting qualitative research and a background in health services research. At the beginning of each focus group or interview, the background of the researcher (no professional expertise or personal experience of the devices) was shared and the reasons for conducting the research. Only participants and JH were present during the focus groups and interviews. JH met patient and carer participants prior to their participation.

**Table 1:** eMMD devices used in focus groups

Lifemax Pill Box Reminder	Pendant design timer with 2 pill compartments. Alarm sound and light to remind when medication is due http://www.lifemaxuk.co.uk/hearing/pendant-pill-box-reminder.html
Lifemax Vibration 5 Alarm Pill Box	Medication planner with 5 pill compartments each with a vibration and/ or sound reminder alarm http://www.lifemaxuk.co.uk/hearing/5-times-a-day-pill-box-reminder.html
Medsignals	Programmable for up to 4 drugs or doses. Rests on a cradle that plugs into telephone and electrical lines to allow daily uploading of dosing history to host server and recharging of battery http://www.medsignals.com/
Medfolio	28 compartments, sends visual, audio, email and text message reminders. Includes visual portfolio of all medications. Medication dosing events communicated to online cloud server https://www.medfoliopillbox.com/
Pivotell Advance GSM Automatic Pill Dispenser	28 compartments and reminds user by alarm and flashing light. Only current dose available at any one time. Can be programmed to send text or email message to carer if medication not taken http://www.pivotell.co.uk/Pivotell%C2%AE+Advance+GSM+Automatic+Pill+Dispenser/0_CAAA001/PRAA002.htm
NRS Med-E-Lert Dispenser	28 compartments and reminds user by alarm and flashing light. Only current dose available at any one time https://www.nrshealthcare.co.uk/household-aids/medication-management/medelert-spare-tray-key
E-Box	Contains pouches which are filled by robot in the pharmacy, supports up to 28 days medication. Only current dose available at any one time. Reminds user by alarm and flashing screen. Dosing events recorded onto website and can alert carer if medication not taken http://robotiktechnology.uk/community-pharmacy/
DoPill (note: despite extensive efforts it was not possible to obtain the device and it was necessary to use a picture of the device as a prompt)	28 compartments and reminds user by alarm and flashing light at each compartment. Can be programmed to alert carer if medication not taken or wrong medication taken https://www.telushealth.co/products/pill/

### Data management and analysis

All recordings were transcribed and the transcripts checked against the recording. Transcripts were not returned to participants for checking. They were analysed thematically based around a framework of the research questions. Coding of the transcripts was conducted using NVivo V.10. Constant comparison within and between cases was used to ensure the analysis represented all perspectives and negative cases were sought for each code. Emergent findings were discussed and checked with the wider research team (n=5) and the coding reviewed and refined.

## Results

Forty professionals (11 pharmacists, 9 GPs, 12 community nurses and 8 social care managers) participated in six focus groups (n=35) and four interviews (n=5). Professional characteristics are in tables 2 and 3. Of 340 patients approached, 24 expressed an interest in the study (7.1%). Fifteen patients were willing and eligible (15/24, 62.5%), and participated in one of two focus groups (n=9) or an interview (n=6). One carer participated in a focus group and two were interviewed. Patient characteristics are in tables 4 and 5. All patients were already using an MMD or eMMD. Focus groups and interviews were each ∼1 hour in duration. Professional, patient and carer characteristics are shown in tables 4 and 5.

**Table 2:** Professional participant gender and age

Professional group	Gender Female (%):male (%)	Age Median years (range)
Pharmacists	64:36	45 (35–58)*
General practitioners	67:33	56 (44–62)
Community nurses	83:17	39 (31–56)
Social care managers	63:37	48 (34–57)†

*Missing for three participants.

†Missing for four participants.

**Table 3:** Professional participants levels of practice deprivation

ID	Levels of deprivation^22^
PH01	10
PH02	10
PH03	5
PH04	4
PH05	8
PH06	6
PH07	1
PH08	10
PH09	6
PH10	9
PH11	9
GP01	7
GP02	6
GP03	4
GP04	4
GP05	4
GP06	1
GP07	4
GP08	4
GP09	4

Scores are from 1 (highest levels of deprivation) to 10 (lowest levels of deprivation).

Note: community nurses and social care managers covered geographical area containing a multiple levels of deprivation 1–10.

**Table 4:** Patient and carer participant gender and age

	Gender Female (%):male (%)	Age Median years (range)
Patients	73:27	73 (43–92)
Carers	100:0	60 (55–62)

**Table 5:** Patient characteristics

	Morisky 8-item Medication Adherence Questionnaire	MMD or eMMD user		Bailey Lovie Reading Test, British ‘N’ system	Rolyan 9-hole Peg Test, dominant hand	Rolyan 9-hole Peg Test, non-dominant hand
ID		MMD	eMMD			
P01	0	X		5	30	39
P02	3	X		3	24	26
P03	1	X		5	22	23
P04	0	X		5	21	43
P05	2	X		6	28	32
P06	2	X		8	24	Unable to complete due to stroke
P07	7	X		5	23	25
P08	3	X		6	20	26
P09	3	X		3	20	26
P10	4		X	4	37	29
P11	3	X		8	27	29
P12	6		X	2.5	23	25
P13	3		X	50	33	34
P14	5		X	6	28	22
P15	3	X		6	26	28

Morisky 8-item Medication Adherence Questionnaire: >2=low adherence; 1 or 2=medium adherence; 0=high adherence.

Bailey Lovie Reading Test, British ‘N’ system: participant is asked to read sequence of words in increasingly smaller print. N ranges from 2 (smallest print) to 80 (largest print). Score indicates smallest print where all words in line accurately read.

Rolyan 9-hole Peg Test: time (in seconds) it takes to individually place the pegs into the board and then remove them.

eMMD, electronic multicompartment medication device; MMD, multicompartment medication device.

Three overarching themes were identified and these are listed in table 6 along with their subthemes.

**Table 6:** Thematic coding framework

Overarching themes	Subthemes
Patient-related issues	Patient characteristics Medication regimen Personal and social circumstances of patients and their carers
Device-related issues	Physical properties Carer's alert
Professional-related issues	Responsibilities Regulatory issues

10.1136/bmjopen-2016-012915.supp1 supplementary appendix

### Patient-related issues

#### Patient characteristics

There was consensus between patient and professional participants that those most likely to benefit from an eMMD were patients with mild memory loss.

I think people who have maybe got memory difficulties, but otherwise relatively intact, cognitively, would probably do quite well. The alarm would remind them, they would understand what was coming out, and how to deal with it.

(GP08)

Some patient participants who were current eMMD users reported using the device due to difficulty remembering to take their tablets.

Well I realised I was forgetting to take my tablets, only because I felt terrible…So my memory is terrible. So that [eMMD] reminds me to take my tablets. (P12, current eMMD user)

Patients using a conventional MMD varied in their response as to whether an eMMD would benefit them personally. Some recalled occasions when they had forgotten medication and thought a prompt would have been helpful whereas others reported they did not usually forget medication. This latter view was echoed by professionals that MMD users tend to be confused about their medication regimen (what to take and when to take it) and are not necessarily forgetful.

We generally bring these systems in, Dosette boxes, when people are confused about their medication, not necessarily forgetful…So a reminder might be infuriating to them but it's not enough…they need instruction…from a person. (GP07)

Nearly all participants were in agreement that eMMDs were unlikely to be suitable or acceptable for patients with more severe memory loss or dementia and professionals reported negative experiences using eMMDs in that population.

I think both patients had memory deficiency, and just couldn’t cope with it on their own. One ended up smacking it with a hammer to stop it, to stop it bleeping. (PH01)

A number of professionals thought people with mental health problems who tend to have poor medication adherence could potentially benefit from eMMD prompts. Similarly, some thought that people with a learning disability could benefit if appropriately supported.

I certainly had one mental health patient, where the electronic device was very good…they used to beat themselves up if they’d missed a dose, and take all their medication as punishment. Whereas, with the electronic device they couldn’t do that, because it was locked in, and it obviously prompted them to regularly take the medication. (PH09)

You could imagine someone with a learning disability getting quite sort of positively focused on it…So they could be taught and encouraged to respond to the alarm. (GP07)

There was broad agreement that people needed to have a good level of manual dexterity and visual acuity. However, devices with an audible prompt could be helpful for patients with sight impairment.

And almost every device requires you to have manual dexterity, so they have to be fairly physical fit, or have the dexterity to use them. (GP09)

With this in mind, participants felt the device would need to be matched to the patient.

It may be that one is perfect, the perfect device for one patient…another one is perfect for the other. So ease of use is very subjective to the person who's using it. (GP06)

It was perceived that elderly people would struggle with an eMMD although looking forward, increasing familiarity with technology may impact on usability in this age group.

It's not a group of people who are particularly comfortable with technology…that in itself, might be something of a barrier…if you’re thinking about future proofing some of these things, the people who are starting to get older now are…more technologically comfortable, than perhaps people who are very old at the moment. (GP08)

#### Medication regimen

Accounts among current or previous eMMD users suggested personal alerts could be beneficial for stable chronic medicines.

…I've got antidepressants in there. If I forget to take them…it's not a nice time, so it reminds me to take them and painkillers. If I didn't take the painkillers, I would be in a lot worse pain…So the benefits are it reminds me to take the painkillers so I feel better. (P012, current eMMD user)

Professionals identified a number of other timing issues, for example, avoiding taking all medication at the same time, once weekly medication and coordinating the timing of two different medications. Some participants felt that a carer's alert could be beneficial when it was vital to a patient's well-being that they took their medication.

…If you have something, some medication, you're going to be ill if you don't take, or whatever…it's [personal reminder] a very good thing to have. (P15, MMD user)

When professionals were handling the devices most commented that patients likely to be using an eMMD would be taking multiple medications. However, opinion differed regarding the number of medications and was dependent on the physical properties of the devices (see ‘Physical properties’ section). Talking about this issue, one current eMMD user said:

I take about ten tablets a day…So it's ideal for people who take about more than five tablets a day…because you can't remember what you've taken. (P13, eMMD user)

Other issues raised were in relation to beginning a new medication regimen or where it was important they take the full course.

People quite often forget to take their antibiotics and if you had a…dose for a couple of weeks that would be really good. (CN11)

#### Personal and social circumstances of patients and their carers

Patient and professional participants thought that eMMDs would benefit people living alone and supported to live independently.

That [eMMD] would be fantastic…if you're in every day and you're not going anywhere…Like, when I came home from hospital, I wasn't able to move, but I should have stayed in hospital, but I didn't. I was like, I want to get home. (P12)

I think these would be good for people who live alone. (P11)

Somebody that's living by themselves that you are supporting to live as normal a life as possible. (PH07)

In contrast, many did not regard an eMMD as an adjunct to carer visits where other systems appeared to work in practice and had additional benefits of human contact.

If they’ve got carers, then we don’t need to be doing down the route of these…there's other more, easier channels to use, like the MAR charts and things. (PH03)

A lot of people who are frail and elderly, living at home, do benefit from having that little bit of contact with somebody on a regular basis…I think compliance is going to be better if you've got actually a person talking to you. (GP07)

Where carers/family were living with a person using an eMMD, professionals suggested it may facilitate carer to leave the house for work or socialising.

I imagine somebody looking after an elderly relative, living with them but going out to work could well say, now there're your breakfast tablets; now the machine's going to go off at lunchtime, when it goes off take your tablet. (GP07)

### Device-related issues

#### Physical properties

Participants generally rated smaller and lighter devices more favourably (Lifemax 2 compartment, Lifemax 5 compartment and Medsignals) particularly for the ability to take the devices out of the home environment. Larger and/or heavier devices would not be practical to take out of the home (E-Box, Medfolio and Pivotell). Devices with a familiar design (to current MMDs) were also favoured (DoPill and Medfolio). The compartments and pouches to store medications were frequently found to be difficult to open or operate, or anticipated they would be for other people (DoPill, E-Box pouches, Lifemax 5 compartment, Medfolio and Pivotell). In addition, the size of each compartment was of concern for professionals, who thought many were too small to accommodate multiple medications (Lifemax, Medfolio and Pivotell).

If you have reasonable doses of medication…they work…But if someone is on a higher level of medication…the carousel jams, because the pills get stuck in it. (SC08)

Some devices had restricted access to the stored medication, only allowing patient access to the dose due to be taken (E-Box, NRS Med-E-Lert and Pivotell). This could be an advantage to avoid taking the wrong dose/overdose; however, it was also disadvantageous to flexibility about when and where to take their medication.

I says, so I've got to carry that about with me if I go out? ‘Cause all my medication's in there…tying you to the house unless you had spare medication about. (P12, current eMMD user)

#### Carer's alert

Participants suggested a carer's alert may be useful depending on the medication involved (see ‘Medication regimen’ section) and reassuring for patients.

I know it's easier said than done, sort it out and that would take the pressure off them because they [patients] would know, hopefully that somebody would come. (P05)

In contrast, an alert to carers could be annoying for patients who choose to vary timing of medication (false alerts).

I think it would be very irritating for the patients if…they're aware of what they're doing. So they'll start to know which their tablets are and be a bit selective…and this night they take their sleeping tablet two hours earlier and stuff like that. (GP01)

Responsibility for monitoring the alert system was of concern for all participants. All participants questioned the capacity and practical challenges of paid carers to take on the responsibility.

So I think it sounds great in practice…but the realities of…getting a carer back in a reasonable time to deal with that issue, would be an extremely large workload…on an already stretched social services. (PH01)

Professionals reported their services could not support this type of monitoring and thought family and informal carers would need to take on the responsibility to receive alerts. However, participants questioned the capacity and capability of family and informal carers to take on the additional duty.

If we were giving that out, I think I would only be giving it out to families who were willing to commit. (CN01)

Professionals suggested that an alert could be used to establish patterns of adherence that would not require daily monitoring. These could be collected centrally and shared with carers and professionals.

### Professional-related issues

#### Responsibilities

There was widespread concern among all participants as to who would be responsible for filling the device, programming the alarm settings, to deal with any problems that occurred and to pay for them. There was little consensus regarding who would take on these responsibilities. Not all patients and carers thought that pharmacists would be prepared to fill a device and that they themselves would find complex devices challenging.

I wouldn't be able to set it up digitally, because I saw him once opening it up in front of me and it's all these sections for your pills. (P10, eMMD user)

Pharmacists said they would not be prepared to fill and programme the devices due to workload concerns and this view was echoed by many other professionals.

We had a lot of concerns over them, because who is responsible for the batteries…Who is going to fill them up? There was all the issues with the pharmacists in the past. (SC04)

Many professionals thought that the responsibility for filling and programming the devices would have to rest with the patient's family. A family member who was a carer could learn how to use a device but might require education/training in the first instance. However, they recognised it could be challenging for family members to do so and reported previous experience where family members were reluctant to take on this type of responsibility.

Many professionals were also concerned about who would be responsible if problems occurred with the device. Professionals who had experience of using eMMDs described difficulties in resolving problems when a device was faulty.

From a pharmacy point of view…they’re a bit of a nightmare to fill and look after. Because you don’t really know where they’ve been, and until you open it, you don’t know what the situation is inside it. (PH08)

Participants perceived the devices to be expensive and raised concern about purchase and implementation costs. Other costs, including a fee for filling a device and training needs for those involved were also highlighted as areas of concern.

I don't imagine that you would get a Council, or a health board…paying for these to be rolled out anywhere. Most of these kind of things that are a bit of a help you say to patients, well this is an option but you have to go and get it yourself. (GP01)

#### Regulatory issues

A number of pharmacists raised concerns that many of the devices do not meet labelling requirements, are not able to carry all the relevant details about the medications and medications are not sealed in the devices. There were also generic concerns about the removal of medication from original packaging and its effect on drug stability. Some medications would not be suitable to include in an eMMD such as unstable medications like hygroscopics, medication which require to be dissolved and drugs whose dosage change frequently like warfarin. Knowing which drugs were inappropriate to store together would require training for whoever was filling the device.

The problem with these is, they don’t meet pharmacy requirements, to enable to seal the tablets into the security capsule labelling requirements. And be able to access the relevant information onto the back of the cell. (PH01)

## Discussion

Patients, carers and health/social care professionals agreed that eMMDs which prompt or remind the patient to take medication may be beneficial for selected individuals, particularly those with cognitive impairment, but who are not seriously impaired, provided they have a good level of dexterity. eMMDs may also assist individuals who are being supported to live alone and require to take medicines at fixed time intervals. Participants who used eMMDs scored high on dexterity and low on adherence but were not elderly. No single device suited everybody; smaller and lighter devices were preferred but their usefulness was limited by the small number and size of storage compartments to accommodate multiple medications. Operating and removing medications from the devices was often challenging. Transportability was a consistently important factor for patients and carers. A carer's alert if medication is not taken was problematic with multiple barriers cited to implementation and no consensus as to who should receive the alert. There was a particular lack of enthusiasm among professionals amid concerns about responsibility for filling, programming, maintaining and funding devices as well as ensuring devices met regulatory standards for storage and labelling of medication.

The strength of this study lies in the inclusion of a wide range of stakeholders with experience of either eMMDs or MMDs. The opportunity to handle and use the eMMDs ensured the data were based on actual experiences and contemporaneous feedback on available eMMDs. The study has a number of limitations. Carers were under-represented; our method to identify carers was through the recruited patients but few patient participants had an informal carer. A further limitation was that the majority of the participants were professionals rather than patients and carers. Although typical for focus group research, a further limitation was the low response rate among all stakeholder groups, especially the patients. Despite this, the spread of demographics for the focus groups likely captured a range of views on the topic. Patients and carers were from 44 to over 90 years of age and self-reported medication adherence ranged from low to high. Professionals were drawn from pharmacies in areas with differing deprivation levels as well as including a range of ages and balanced gender mix. Finally, patient participants were already MMD or eMMD users and the findings from this study may not be applicable to patients who have adherence problems but do not use one of these devices.

We are not aware of any directly comparable studies to this one. Recent trial data of personal reminders from an eMMD and text messaging in patients with tuberculosis reported problems that occurred with the eMMDs.^21^ These were most commonly incorrect use by the patient, failure of the device and battery problems and often necessitated replacement of the device. Despite these issues, non-adherence was reduced; however, the population was younger than participants in this study as well as being from a predominantly rural (farming) location with low levels of education and findings may not be applicable to other populations. In contrast, McGillicuddy *et al*^27^ reported high levels of satisfaction and usefulness among patients with kidney transplant with a system of eMMD and text message reminders. However, this was a small-scale proof of concept study where the carer's alert function was received by the study coordinator, not the clinician. The clinician responsible for patient care received a weekly report of patient adherence that was tailored to their preferences. Findings from these studies cannot be translated to the generic polypharmacy population. In addition, they do not address the issue of transportability of an eMMD that was consistently raised by patients in this study.

This study shows that eMMDs would need to be carefully targeted at those community-based individuals most likely to benefit. The choice of device would depend on the individual and be matched to their specific needs. Consideration of size, weight, capacity, security and transportability are likely to be important issues for consideration when selecting a device. Personal reminders to take medications seem to be acceptable but carer's alerts may be more problematic. The success of this type of monitoring will depend on the ongoing agreement of a person (or organisation) to receive the alert as well as consistency and clarity in the subsequent response to the patient. Professionals may be unlikely to support this function and may depend on families who have both the capacity and capability to take on this responsibility.

Of greater concern was the general lack of enthusiasm among professionals and pharmacists in particular. For eMMDs to be used successfully, detailed planning with all parties and clear agreement with whoever agrees to take on responsibilities for filling, programming, maintaining and paying for the device and its operation would have to be agreed. Without such agreements in place, successful implementation is unlikely. Concerns about safe storage of medications may also be challenging to overcome with some of the devices used in this study.

In conclusion, this study provides indicators of which patients are likely to benefit from an eMMD as well as the kinds of device features to consider when matching a patient with an eMMD. It also highlights other considerations for successful implementation including issues of responsibility and regulation along with a fee structure to recognise the additional workload for pharmacists. These findings reflect the complexity of non-adherence and caution should be employed in rolling out the use of eMMDs more widely without further research. The effectiveness and cost-effectiveness of eMMDs remains unclear and future research should focus on robust research methods to address these matters, taking account of the findings from this study to inform the research design.
