# Supplementary material for: Views of patients and professionals about electronic multicompartment medication devices: a qualitative study
Source: BMJ Open. 2016 Oct 17;6(10):e012915. doi: 10.1136/bmjopen-2016-012915 (PMC5073531; doi:10.1136/bmjopen-2016-012915)
Supplement: supplementary appendix [file bmjopen-2016-012915supp_appendix.pdf]

**Appendix 1. Topic guides.**

**Patient focus group topic guide**

---

**Introduction and Ground Rules**

**Icebreaker**

**Tell us about your experiences of MMDs and eMMDs?**

**"Show and tell"**
Show the eMMD devices and allow the participants to handle for 5-10 mins.

*using 'think aloud' technique prompt*

- How usable do you think the devices are?
- What do you think are the good points of these devices?
- What do you think are the bad points of these devices?
- How useful do you think the carer's alert is?

**We are planning on doing a bigger study on these devices, it would a randomised controlled trial. This means that half of the people in the study would get an electronic MMD and the other half would get a regular MMD. Which group people would be in, would be decided at random, like the toss of a coin.**

- Do you think you would like to be in a study like this?
- If not, what would you not like?
- How would you feel if you were in the "control" group (MMD)

**If we do a bigger study, we will need to use different measures to see how good they are at reminding people to take their medication. What do you think would be a good measure?**

Prompt

- symptom questionnaires
- pill counts
- results of blood tests
- looking at medical records
- looking at visits to GP and hospital

**If we do a bigger study, what do you think would be the best way to invite people to be part of the study?**

Prompt

- Letter from pharmacist?
- Poster at pharmacist?
- Letter from doctor?
- Some other method?

**End the session:**

- Is there anything else you would like to add?
- Thank the participants

**REMIND Professional focus group schedule**

---

**Introduction and Ground Rules**

**Icebreaker**

**“Show and tell”**
Show the eMMD devices and allow the participants to handle for 5-10 mins.

**Tell me about your experiences of using MMDs and eMMDs**

- Perceived advantages, perceived disadvantages, acceptability.

**What are the advantages and disadvantages of different means of contacting carers when adherence problems are detected?**

**What are the advantages and disadvantages of adherence measures such as pill counts?**

**In your experience who usually makes the decision to use MMDs in general?**

- doctor, nurse, patient, lay carers, pharmacist

**Why do some patients receive these aids on the NHS while others do not? How many people purchase their own system?**

**Which people are most likely to benefit from eMMDs**

- how those patients can best be identified using existing tools

**Which conditions  may act as good models for measuring the impact on adherence of these devices**

**What sorts of physiological, clinical or biochemical outcomes might be practical to use in a RCT as an outcome measure?**

**End the session:**

- Is there anything else you would like to add?
- Thank the participants
